# Perspective of Immunopathogenesis and Immunotherapies for Kawasaki Disease

**DOI:** 10.3389/fped.2021.697632

**Published:** 2021-07-19

**Authors:** Lung Chang, Horng-Woei Yang, Tang-Yu Lin, Kuender D. Yang

**Affiliations:** ^1^Department of Pediatrics, MacKay Memorial Hospital, Taipei, Taiwan; ^2^Division of Infectious Disease, MacKay Children's Hospital, Taipei, Taiwan; ^3^Department of Medical Research, MacKay Memorial Hospital, New Taipei City, Taiwan; ^4^Department of Medicine, Mackay Medical College, New Taipei City, Taiwan; ^5^Division of Allergy-Immunology-Rheumatology, MacKay Children's Hospital, Taipei, Taiwan; ^6^Department of Microbiology & Immunology, National Defense Medical Center, Taipei, Taiwan; ^7^Institute of Clinical Medicine, National Yang Ming Chiao Tung University, Taipei, Taiwan

**Keywords:** coronary artery aneurysm, coronavirus disease 2019, immunotherapy, intravenous immunoglobulin resistance, Kawasaki disease, Kawasaki disease shock syndrome, multisystem inflammatory syndrome in children, Th17/Treg imbalance

## Abstract

Kawasaki Disease (KD) is an acute inflammatory illness that mostly occurs in children below 5 years of age, with intractable fever, mucocutaneous lesions, lymphadenopathy, and lesions of the coronary artery (CAL). KD is sharing clinical symptoms with systemic inflammatory syndrome in children (MIS-C) which is related to COVID-19. Certain genes are identified to be associated with KD, but the findings usually differ between countries and races. Human Leukocyte Antigen (HLA) allele types and toll-like receptor (TLR) expression are also correlated to KD. The acute hyperinflammation in KD is mediated by an imbalance between augmented T helper 17 (Th17)/Th1 responses with high levels of interleukin (IL)-6, IL-10, IL-17A, IFN-γ, and IP-10, in contrast to reduced Th2/Treg responses with lower IL-4, IL-5, FoxP3, and TGF-β expression. KD has varying phenotypic variations regarding age, gender, intravenous immunoglobulin (IVIG) resistance, macrophage activation and shock syndrome. The signs of macrophage activation syndrome (MAS) can be interpreted as hyperferritinemia and thrombocytopenia contradictory to thrombocytosis in typical KD; the signs of KD with shock syndrome (KDSS) can be interpreted as overproduction of nitric oxide (NO) and coagulopathy. For over five decades, IVIG and aspirin are the standard treatment for KD. However, some KD patients are refractory to IVIG required additional medications against inflammation. Further studies are proposed to delineate the immunopathogenesis of IVIG-resistance and KDSS, to identify high risk patients with genetic susceptibility, and to develop an ideal treatment regimen, such as by providing idiotypic immunoglobulins to curb cytokine storms, NO overproduction, and the epigenetic induction of Treg function.

## Infection Versus Autoimmunity in Kawasaki Disease

Kawasaki Disease (KD), previously known as mucocutaneous lymph node syndrome was first reported by *Tamisaku Kawasaki* in 1974 (1). KD is a systemic inflammatory disease complicated with medium-sized vasculitis that is found mostly in children younger than 5 years of age, with at least four out of the five clinical features: pleomorphic skin rashes, bilateral non-purulent conjunctivitis, changes in oral mucosal: dry fissured and erythema of lips, and strawberry tongue, changes in the appearance of peripheral extremities: indurative angioedema of the hands and feet, followed by skin desquamation, and cervical lymphadenopathy (at least 1.5 cm in diameter) (1–3). Pathogens such as *S. aureus*, streptococci, coronavirus, enterovirus, Epstein-Barr (EB) or rhinovirus virus had been suspected to be associated with KD (4–8). In East Asia, Bacillus Calmette-Guérin (BCG) vaccination is a standard procedure into infants. There, about 40% of the KD patients develop a reactive skin erythema and scaling at the site where BCG was inoculated. This suggests that the reaction at the BCG inoculation site is related to a cross-reaction of BCG antigen or a bystander of the hyperinflammatory reaction of KD (9). Asymptomatic infection may lead to KD in children with an underlying genetic predisposition (10). However, no invariable single pathogen readout in the sera from patients of KD has been observed (11). Although siblings of KD patients have a six to 10 times higher risk developing KD than those without a family history, there is no evidence of a contagious transmission of KD in day care centers or among hospitalized patients (12, 13). It is debatable whether KD is a post-infectious hyperinflammation reaction, an autoinflammatory syndrome or an autoimmune disorder (14–16). Inflammation-inducing substances may play an important role in KD, such as those originating from pathogens, pathogen-associated molecular patterns (PAMPs), toxins (superantigens), and antigens from injured or infected-host cells which behaved as damage-associated molecular patterns (DAMPs) that are causing the hyperinflammatory response of KD (17).

In 2005, a novel human coronavirus named human coronavirus New Haven (HCoVNH) is reported to be linked to a cluster of KD in New Haven, by evidence of positive RT-PCR detection of 8/11 vs. 1/22 in a case-control study (7). This correlation between KD and HCoVNH is not observed in Taiwan. Viral RNA of HCoVNH or HCoV-NL63 in nasopharyngeal secretions is not detected among 53 consecutive KD subjects (8). Recently, during the pandemic of coronavirus disease 2019 (COVID-19), more than 1,000 cases of KD-like multisystem inflammatory syndrome in children (MIS-C) have been reported (18). MIS-C shares similar symptoms with KD, such as fever, skin rash, conjunctivitis, mucous membrane involvement. Some MIS-C patients even meet the full diagnostic criteria for KD (19). Over 80% of the patients are positive in serological test for anti-severe acute respiratory syndrome coronavirus 2 (SARS-CoV-2) spike protein antibodies, but <30% of them had detectable viral RNA in upper respiratory tract (18, 20–24). The immunologic markers of KD and MIS-C are also overlapping, as such inflammatory cytokines of interleukin (IL)-1, IL-6, IL-8, and IL-17 are usually elevated. The fact that MIS-C occurs after SARS-CoV-2 infection presenting a KD-like disorder suggests that both KD and MIS-C have similar pathogenesis of autoimmune etiology induced by certain viral infection (25). Therefore, the algorithm for diagnosis and treatment of typical and atypical (incomplete) KD in children has been adopted for the early recognition and management of the MIS-C, such as treatment with IVIG and corticosteroids (26). Interestingly, the MIS-C cases are mostly reported from Italy, France, UK, and USA but not found in Asia, where the incidence of KD is 10 times higher than that in Western countries (24, 27). It is worth to mention previous reports of increasing KD incidences after hepatitis B and influenza vaccinations (28–30). There is also evidence of autoimmune disease flared up after SARS-CoV-2 vaccination recently (31). Now the COVID-19 vaccines have been widely applied to young adults and old adults but not young children. It deserves our concern whether COVID-19 mass vaccination in children might increase the MIS-C. It is reasonable to concern that the incidence of MIS-C might increase after the extensive vaccination against SARS-CoV-2, in which the cross-reactivity of T lymphocytes and vaccine antigen in the presence of adjuvants might raise the risk of autoimmune vasculitis (32). These observations suggest that children in Western countries are more susceptible to coronavirus-related KD-like vasculitis, which apparently has a different etiology from the KD vasculitis in the Asian population.

## Immunopathogenesis of Kawasaki Disease

Various infectious disease cause mucocutaneous rashes in combination with neutrophilia and elevated C-reactive protein (CRP). However, KD is not contagious, the vasculitis is regarded as the result of hyperinflammatory reactions which are attributed to immune responses (14, 16). Patients with KD are presenting with an increased T helper cell 17 (Th17) count, diminished regulatory T cell (Treg) reaction, and a higher neutrophil vs. lymphocyte (N/L) ratio in the KD cases of IVIG resistance and coronary complications. This implied an imbalance between pro-inflammatory and immunoregulatory responses (33, 34).

We first demonstrated that in children with KD, the immune activation marker CD40L is highly expressed on T cells and platelets, and that the overexpression of inducible nitric oxide synthase (iNOS) is associated with elevated nitric oxide (NO) levels in blood samples of KD patients before IVIG therapy (35, 36). These findings indicate that the intensity of innate and adaptive immune reactions is reversed after IVIG therapy. T cell polarization is skewed toward the Th2 pathway as a response to IVIG therapy, a high eosinophil count and elevated IL-5 levels are favorable markers for the success of an IVIG treatment, in contrast, lower initial eosinophil counts, IL-4, and IL-5 cytokine levels are related to a refractory response of IVIG (37, 38). KD patients have prominent Th17 immune responses and diminished Treg pathway transcription factor, FoxP3 expression while compared to a febrile control group without KD (39).

The susceptibility to KD is also related to an alteration of the Treg response in respect to polymorphisms of the transforming growth factor (TGF)-β signaling pathway genes TGF-β2 and SMAD3, which downregulate the Treg immune response (39, 40). Moreover, some KD patients are presenting with a phagocyte activation syndrome due to the overproduction of interferon (IFN)-γ, and its downstream mediators of interferon-induced protein (IP)-10 and tumor necrosis factor (TNF)-α (41). In addition to early neutrophilia, KD patients are usually suffering from thrombocytosis in later stages of the disease (42). Taken together, these studies indicate that the kinetic immune responses in KD consists of an early augmentation of the innate immunity, follow by an imbalance between Th17/Th1 and Th2-Treg responses of the adaptive immunity. This assumption is consistent with observations made in autopsies, showing early necrotizing vasculitis with innate phagocyte activation is followed by the remodeling of coronary thrombosis with lymphocyte infiltration and the formation of aneurysms in the adaptive immunity phase (43, 44). These findings also suggested that a comprehensive immunotherapy may be required to modify the immunopathogenesis of KD, depending on the progression of the disease.

## Immunogenetics and Clinical Phenotypes of Kawasaki Disease

KD can be categorized into different phenotypes according to clinical presentations and the severity of the disease (Table 1). Typical KD presents as a systemic vasculitis with fever of more than 5 days and meets the aforementioned clinical features. Patients with the typical KD criteria but fever <5 days may be diagnosed with KD if coronary aneurysm, dilatation and/or lesion is found. Children with prolonged fever and suspected of having KD but not fulfilling the complete diagnostic criteria are regarded as incomplete or atypical KD. Children with <6 months or more than 5 years of age tend to have atypical KD, which usually is associated with delayed in diagnosis and treatment (45, 46). These atypical KD patients tend to have a high risk of IVIG resistance and coronary arterial abnormalities (45, 47, 48). Adults with coronary lesions are often contemporarily or retrospectively diagnosed with atypical or incomplete KD (49, 50).

**Table 1:** Different phenotypes and characters of Kawasaki disease.

**Phenotypes**	**Kawasaki disease (KD)**		**KD shock syndrome (KDSS)**
	**Typical KD**	**Atypical KD**	
Criteria	≧4/5	<4/5	≧4/5 + shock
Myocarditis	5%	20%	20%
Age (year) (avg)	0.5–5.0 (2.0)	Frequent <0.5 or >5	2–12 (3.5)
Platelets (1,000/ul)	>350	>450	High or low
WBC (/mm^3^)	>10,000	>15,000	Variable
Pyuria	Some	Frequent	Frequent
CRP (mg/dl)	3–15	>3	>10
Procalcitonin (ng/ml)	>0.5	Variable	>1.0
Ferritin (ng/ml)	100–200	100–200	>500
Coagulopathy	No	Some	Often
D-dimer (ng/ml)	<1,000	<1,000	>1,000
Cytokines	IL-6, IL-10, IP-10	IL-6	IL-6, INF-γ, IL-10
IVIG Resistance (%)	15	>15 (delayed Rx)	40
Fatality (%)	0.1	Unknown	2

*WBC, white blood cell; CRP, C-reaction protein; IL, interleukin; INF, interferon; IP, interferon-γ induced protein; IVIG, intravenous immunoglobulin*.

A severe form of KD complication with life-threatening myocardial dysfunction and hypotension is known as KD shock syndrome (KDSS). According to a population-based study in Taiwan, KDSS patients are older and higher risk of coronary artery complications (51). As shown in Table 1, levels of CRP and procalcitonin are elevated in the KDSS group. Ferritin (>500–1,000 ng/ml) and D-dimer (>1,000–4,000 ng/ml) are also significantly higher in children with KDSS (52–54). The inflammatory mediators, phagocyte activation and coagulopathy profiles are very similar between KDSS and MIS-C (26). In general, hyperferritinemia in KDSS is thought to originate from macrophage activation syndrome (MAS), and thus is concurrent with hypertriglyceridemia, anemia, and hypercytokinemia. Moreover, the pathogenesis of the shock syndrome in KD may be explained by the overproduction of nitric oxide. KDSS is more frequently taking a complicated course with pulmonary symptoms, acute kidney injury (AKI), pancreatitis, hepatitis, and neurological disorders (53, 55, 56). The systemic cytokine storms in KD and KDSS are similar, with hypercytokinemia of IL-6, IL-10, IL-17, IP-10, and monocyte chemoattractant protein (MCP)-1. However, the levels of IL-6 and IL-10 are significantly higher in KDSS (54, 57). There is a risk of thrombosis in KDSS patients who are frequently associated with lower platelet counts, and coagulopathy (52, 58). As the incidence of KD and KDSS differs widely among ethnicities and countries, a contribution of environmental factors and the diverse genetic background to different clinical phenotypes of KD may be assumed.

## Ethnical Differences and Genetic Susceptibility of Kawasaki Disease

KD is prevalent in many East Asian countries, e.g., in Japan, China, Korea and Taiwan (2, 17, 59, 60). For children younger than 5 years, the highest incidence has been observed in Japan (more than 250 cases per 100,000 children). In India, the incidence is the lowest (only 4.5 per 100,000 children) (61, 62). Children of Japanese ethnicity have the highest incidence of KD in Hawaii, before other Asians, Chinese, and Caucasian children (61). Several genes have been linked to the susceptibility of KD, e.g., B-lymphoid tyrosine kinase (BLK), cysteine-aspartic acid protease (CASP)3, CD40, Fc fragment of IgG receptor IIA (FcγRIIA), inositol-triphosphate 3 kinase C (ITPKC), and calcium release-activated calcium channel protein (ORAI)1 (63–65). Human leukocyte antigen (HLA)-BW22J2 is prevalent in the Japanese population but not in Caucasians (66). In Korean children, the frequency of HLA-DRB1^*^11 is a risk factor for KD with coronary artery lesions (CAL), HLA-DRB1^*^09 has been shown to be protective (67). In Taiwan, the HLA-DRB1 is associated with susceptibility to KD, and the MHC-class-I-chain-related gene A (MICA) A4 in KD is protecting children with KD from developing CAL, which has been validated in a genome-wide association case-control study (GWAS) in the Taiwanese population (68–70). Since the role of HLA is to present antigenic molecules to the immune system, a specific HLA subtype activate T cells and induce a hyperinflammation response depending on the HLA polymorphisms among races and ethnicities (71).

In addition to HLA subtypes, we have discovered that TGF-β2 and SMAD3 variants are associated with KD in the population of Taiwan (40). A dominant T allele of rs2243250 in the IL-4 gene is conferring a significant protective effect against the development of CAL (*p* = 0.006) (72).

In the ongoing COVID-19 pandemic, as an illness that manifests with KD-like features, MIS-C is thought to be a rare complication associated with COVID-19 (18). The atypical KD-like symptoms alert physicians to recognize it early and to adopt an adequate treatment for MIS-C patients. The connection between MIS-C and COVID-19 suggests that post-infectious autoimmune vasculitis depends considerably on the individual genetic background. It is also of concern that the incidence of MIS-C might increase after the extensive vaccination against SARS-CoV-2, where the cross-reactivity of T lymphocytes and the vaccine antigen in the presence of adjuvants might raise the risk of autoimmune vasculitis (32). The contribution of the Th17/Treg imbalance to autoimmunity is different between MIS-C and KD, because the Th17 mediators are elevated in both diseases but the immunosuppressive mediators stem cell factor (SCF), negative regulator of INF-γ (TWEAK), and adenosine deaminase (ADA) in KD are lower than in MIS-C. This suggests that KD and MIS-C patients do require different strategies for immunotherapy (25).

## Epigenetic Factors in the Immunoregulation of Kawasaki Disease

In addition to a genetic predisposition of Th17/Treg imbalance, the epigenetic regulation of DNA methylation on innate and adaptive immune genes is also associated with KD. Patients with DNA hypomethylation on the promoter CpG islands of immune activation genes have the tendency of being susceptible to KD and IVIG resistance (73–76). In the acute stage of KD, most of the changes in CpG island methylation have been identified as hypomethylation (97%) (76). These genes are associated with the mRNA expression of toll-like receptors (TLRs 1, 2, 4, 5, 8, and 9) (74). Similarly, innate immunity genes such as FcγRIIA, IL-10, and S100A8 are reported being hypomethylated in KD patients (74–76). The DNA-hypomethylation is reversed after IVIG treatment, which indicates that epigenetic methylation plays an important role in the pathogenesis of KD.

In the meantime, miRNAs have been disclosed to be an ideal biomarker of KD, this may assist in the differentiation of KD from other febrile diseases, based on miRNAs expression profile at C_T_(miR-1246)-C_T_ (miR-4436b-5p) and C_T_ (miR-197-3p)-C_T_ (miR-671-5p) (77). The changes of miRNA expression associated with Treg cells in KD patients before and after IVIG treatment have been documented (78, 79).

The epigenetic control of Treg cells has been demonstrated mainly *via* FoxP3 expression pathway (80). In addition to the individual genetic background, environmental factors are also contributing to the epigenetic profiles and functional markers of Treg subpopulations (tTreg, pTreg, and iTreg). Hence, studying Treg subsets may potentially help to predict and hints the clues of how to prevent Th17-mediated autoimmune diseases (80, 81). Given the fact that the immunopathogenesis of acute KD appeared to include prominent Th17 cell activation and accompanied by a lower regulatory T cell response which will be reversed after IVIG treatment (39, 80–82), we postulated that the dynamics of Treg populations, which are regulated by genetic and epigenetic modifications, may be considered to be useful biomarkers and potentially therapeutic targets of KD.

## Evolvement of Immunotherapies for Kawasaki Disease

**Figure 1:**
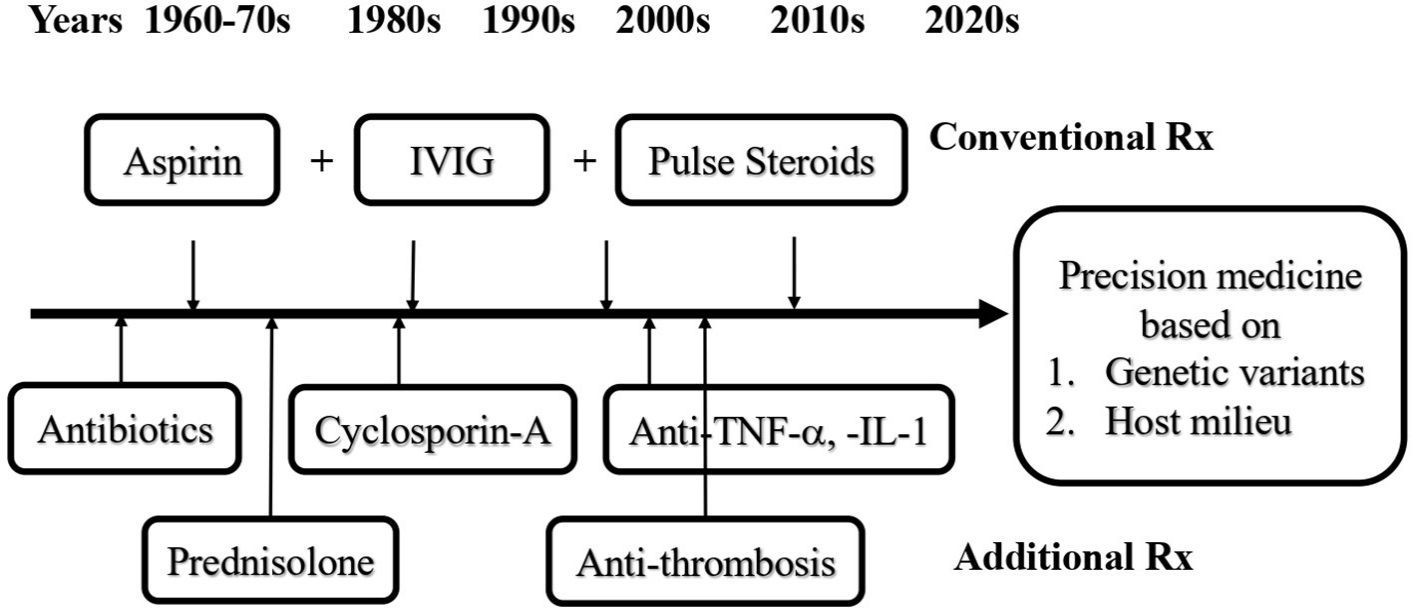
Evolvement of immunotherapies for Kawasaki disease (KD). The conventional treatment (Rx) for KD has been evolved from antibiotics, aspirin, corticosteroids, IVIG and aspirin plus IVIG, aspirin, and/or pulsed corticosteroids through the past 50 years. In KD patients with IVIG resistance or KDSS, additional Rx for refractory KD requires cyclosporin-A, anti-cytokines, and/or anti-thrombotic treatment, depending on the complications. In the era of precision medicine, to screen and to understand the genetic variants and internal milieu of KD patients in advance may help to prevent KD in patients from IVIG resistance or KDSS.

The standard treatment of KD in the acute febrile stage has evolved through the past 50 years, and consists either of a combination of aspirin, antibiotics, and corticosteroids, or of a combination of IVIG, aspirin, and corticosteroids (Figure 1) (1, 26, 83–99). In the 1970's, the era before IVIG therapy, aspirin considered to be a better choice than antibiotics or corticosteroids (1, 83). However, neither high dose (80–100 mg/kg) nor low dose (30–60 mg/kg) of aspirin was able to reduce the prevalence of coronary artery aneurysm (CAA) formation in KD patients (45, 46). After introducing IVIG treatment since the late 1980's, the recommended standard regimen is a combination of aspirin and IVIG therapy. The optimal dosage of IVIG for KD is a single high dose of 2 gm/kg, it turned out to be more effective than protocol of 1 gm/kg/day for 2 days, or 400 mg/kg/day for 5 days (46, 84). Before the IVIG therapeutic regimen had been developed, the incidence of CAA was 25% and the mortality rate was 1–2% in KD patients (1, 2, 83). After that, the sequelae of CAA declined to 3–4% with a mortality of 0.1% (46, 59, 60). While most of the KD patients respond well to the combination of aspirin and IVIG, still 10–20% of the patients are refractory to this therapy (46, 91, 94, 96). In these cases, the treatment with IVIG and pulsed corticosteroids has led to a better clinical outcome (87, 89).

Corticosteroids are commonly considered as a therapy for autoimmune disease. The use of corticosteroids in KD before the era of IVIG had an increased morbidity of CAA at 64.7% vs. only 20% when treated with antibiotics or 11% when treated with aspirin (1, 83). In a meta-analysis, the combination of IVIG with pulsed corticosteroids is found to significantly reduce the risk of CAL compared with IVIG alone (7.6 vs. 18.9%; OR: 0.3; 95% CI 0.20–0.46) (89). In addition to pulsed corticosteroids, KD patient refractory to IVIG may benefit from therapies with cyclosporin, anti-TNF-α, or anti-IL-1 (Figure 1) (93–95, 97). This may be explained by the immunopathogenesis of KD: An augmented proinflammatory cytokine storm in the febrile stage is followed by intensified Treg cell function after IVIG treatment, due to genetic expression and epigenetic modification of immune-regulatory genes. Certain hallmark complications of KD such as KDSS, MAS, or CAA are usually associated with IVIG resistance, suggesting there might be different immunopathological mechanisms present among these patients. Thus, KD patients with underlying various immune phenotypes might require a tailored comprehensive immunotherapy to cease disease progression. Several scoring systems have been developed to predict IVIG resistant KD, e.g., the Kobayashi Score, which is useful for the prediction and prevention of CAL in the Japanese population but shows poor sensitivity and specificity in Western countries (87, 96).

KD patients with IVIG resistance usually present with a protracted course of fever and elevated IL-6 cytokine levels (86). It is reasonable to apply anti-IL-6 therapy for refractory KD patients, because the serum IL-6 level is correlated with IVIG resistance (94). However, in a study of four patients with IVIG-resistant KD showed good response to treatment with anti-IL-6, two of them still developed CAA (95). Some studies have reported the association of high IL-6, IL-10, and TNF-α cytokine levels with refractory KD (46, 86). Lower IL-5 levels and eosinophil counts are also correlated with IVIG resistance (37, 38). Meanwhile, the allele rs2243250T of the IL-4 gene is found to confer protection against CAL (72). KD patients with genetic diversity background may require different criteria to predict a possible resistance to IVIG and individualized anti-cytokine treatment. A detailed scoring system should include clinical symptoms and signs, the individual's immune genotype and laboratory parameters, to guide a precise anti-inflammatory therapy and improve clinical outcomes.

Patients with KDSS are usually suffering from complications of coagulopathy, embolism, and thrombosis, which require anti-thrombotic therapy in addition to IVIG, corticosteroids and inotropic medications (53, 54, 58, 98). The pathophysiology of the coagulopathy is based on host antigen presenting cells (APCs) carrying HLA subtype(s) which interact with PAMPs or DAMPs and induce an innate defense with defensin and type I interferons, followed by an efficient adaptive immunity of Th1 cell immunity and Th2 humoral immunity. Abnormal host-antigen interactions are leading to Th17/Th1; Th2-Treg imbalance and cytokine storm through the expression of IL-6, IL-17A, and IL-10, which manifest in KDSS as systemic vasculitis, thrombosis, and shock. In the era of precision medicine, to correct the imbalance of the Th1-Th17/Th2-Treg response in refractory KD or KDSS, we should not only consider genetic variants, but also HLA subtypes and the host milieu (Figure 1). Homeostasis of vitamins and microbiota may enhance the function of Tregs and suppress the response to cytokines (26, 92, 99–102). For instance, almost all KD patients (98.7 %) had significantly lowered 25(OH)-vitamin D levels (<30 ng/mL) than age-matched non-KD patients (78.6%, *p* < 0.0001) (92). A low level of vitamin D is linked with gut dysbiosis and inflammation in KD with vascular morbidities (99). Therefore, knowing the genetic variants and host milieu of high-risk patients might be beneficial in preventing complications of KD.

## Perspective of Immunotherapies for KD Patients Resistant to Conventional Treatment

**Figure 2:**
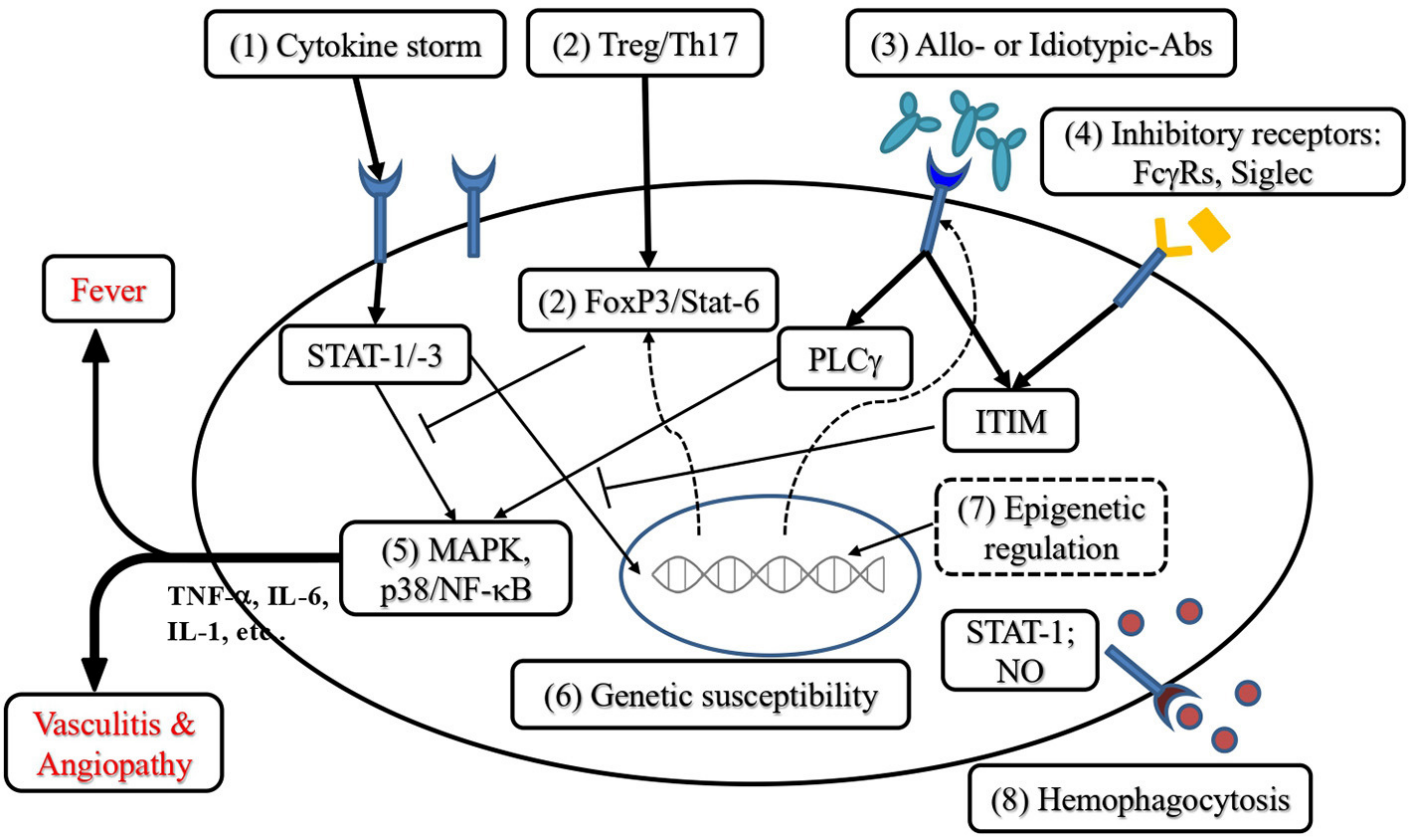
Perspective of immunotherapies for KD refractory to conventional treatment. (1) Targeting the cytokine storm: Anti-TNF-α and anti-IL-1 have been used to rescue IVIG resistance. In addition, other Th17 and Th1 cytokines are also candidates for therapeutic targets. (2) Addressing the Th17/Treg imbalance can be achieved by augmentation of Th2 or Treg polarization, including the enhancement of FoxP3 expression. (3) Blockade or immunoregulation by allotypic and idiotypic IgG provides immunoregulation of KD. (4) Enhancement of immunoinhibitory receptors transduces the immunoreceptor tyrosine-based inhibitory motif (ITIM) to inhibit cytokine production. (5) Targeting the signal transduction of mitogen-activated protein kinases (MAPK) including p38 phosphorylation to suppress cytokine (TNF-α, IL-6, IL-1, etc.) production. (6) Screening genetic variants related to KD with IVIG resistance protects KD patients from coronary complications. (7) Epigenetic regulation of Treg immune responses reverses the Th17/Treg imbalance in KD with IVIG resistance. (8) Anti-hemophagocytosis can be achieved by targeting IFN-γ and TNF-α as well as the inhibition of their downstream effector STAT-1 (signal transducer and activator of transcription 1) or nitric oxide (NO) synthase activation (→ indicates activation; ⊥ indicates inhibitory regulation; - - -> indicates epigenetic regulation).

Based on the understanding of the immunopathogenesis of KD and the resistance to IVIG, we would like to suggest a stepwise approach to IVIG-resistance and KDSS, as described in Figure 2 and below:

1. Targeting Cytokine Storm
  Hypercytokinemia of TNF-α and IL-6 is the hallmark in KD patients with IVIG resistance (103, 104). Thus, some studies demonstrated that anti-TNF-α (infliximab) or anti-IL-1 (anakinra) can provide as an adjunctive therapies for IVIG resistant KD (97). The therapeutic effect of single pro-inflammatory cytokines antagonist based on monoclonal antibody may be suboptimal; a combined regimen or targeting with polyclonal antibodies will be more efficient. Although KD and MIS-C are sharing similar clinical presentations, the Th17 and Th1 mediators Il-6, IL-17A, TNF-α, and IP-10 are more prominently increased in KD than in MIS-C (25, 46, 86). This fact advocates that targeting IL-17A and TNFα should be considered in IVIG resistant KD patients. In contrast to children with MIS-C without IL-17A or TNFα over production, a combination of IVIG and IL-1-receptor antagonist (e.g., Anakinra) might be the preferred therapeutic strategy (25, 105). Acute kidney injury is a serious complication in some KD patients. Particularly, those with KDSS and hypercytokinemia require plasma exchange or continuous renal replacement therapy (56). In critical conditions, extracorporeal membrane oxygenation (ECMO) is indicated for KD patients with life-threatening cardiac dysfunction or hemodynamic instability in KDSS refractory to IVIG, corticosteroids and inotropic agents (106, 107).
2. Targeting Th17/Treg Imbalance
  Genetic and epigenetic alterations in the Treg pathways have been found in patients with KD. The Treg cell development and induction are largely affected by the endogenous milieu, such as vitamins and metabolites of microbiota (101, 108–112). The lower vitamin D levels have been linked to hyperinflammation in COVID-19 and thrombotic complications (111–113). Vitamin D deficiency had been also shown to be associated with IVIG resistant KD and coronary artery complications (92, 110). In addition to vitamin D, gut microbiota coordinates mesenchymal stem cells (MSCs) and relieve experimental autoimmune disorders (114–117). Accumulated evidence suggests that vitamin D supplementation and exosomes derived from MSCs might be useful in treating inflammatory diseases with a cytokine storm (114–116), which might be adopted for the treatment of KD with IVIG resistance.
3. Neutralization or Immunoregulation by Allotypic and Idiotypic IgG
  Despite IVIG remains irreplaceable for its immunomodulatory activity in the treatment of KD, a certain portion (10–20%) of KD patients shows resistance to IVIG (118). The mechanism of IVIG immunoregulatory effect is attributed to neutralizing antibodies, anti-idiotypic antibodies, isotypic/allotypic IgG fractions, and Fc fragments which bind to inhibitory Fc receptors (119, 120). The FcγRIIB is an inhibitory Fc receptor on human phagocytes and B cells with diverse biological functions due to polymorphic variants (121). The genetic variants or isoforms of FcγRIIB can reshape autoimmune disorders, as well as KD (121, 122). Several articles report that maternal anti-idiotypic IgG modulate immunoregulatory phenotypes of B cells and Th17 cells *in utero* or by adoptive cell transfer (123–125). Further efforts should focus on the roles of IVIG antibody fractions and recombinant IgG fragments for the precision treatment of KD.
4. Enhancement of Immunoinhibitory Receptors
  The inhibition of the hyperinflammation could be mediated by the immunoreceptor tyrosine-based inhibition motif (ITIM) through its cytoplasmic tail (126). The ITIM-containing receptors belong to the immunoglobulin receptor superfamily with a consensus sequence of I/V/LxYxxL/V (where x represents any amino acid) and are docking to proteins with SH2 domains, among the protein-tyrosine phosphatases like Shp1 and Shp2 (127). The inhibitory FcγRIIB mediates immunomodulation through an inhibitory ITIM (121, 127). In Caucasian KD patients, polymorphisms of FcγRIIB is related to resistance against IVIG treatment (122). Studies have shown that Fc fragments play a pivotal role in the immunosuppression for autoimmune disorders (121, 122, 128, 129). The Fc domains of IgG isotypes are N-glycosylated at asparagine-297 and 30 N-glycans have been identified and implicated to participate in varying effector functions of IgG (130–132). The N-glycans of the Fc domain with galactosylated glycans are associated with pro-inflammatory diseases; in contrast, the degree of sialogalactosylation on the N-glycosylated Fc domains in antibody preparations are proportional to anti-inflammatory effect (132).
  The CD24Fc is a fusion compound consisting of a Fc domain and a CD24 sialoglycoprotein. It shows a protective effect in an animal model of virus-mediated respiratory inflammation. A commercial version of CD24Fc has been examined in a phase three clinical trial for COVID-19 immunomodulatory treatment (133). The highly sialylated CD24 glycoprotein is able to induce immunosuppression by the ligation of a sialoglycan to sialic acid immunoglobulin-like lectin-10 (Siglec-10), and activates multiple physiological effects, such as mediating a driving force to cancer, damping excessive tissue inflammation, and inducing immune tolerance in pregnancy (134–136). A novel cell membrane-insertable lipid-modified synthetic sialoglycan has been investigated for immunomodulatory properties (137). It inhibits *in vitro* overactivation of neutrophils by extracellular neutropil traps (NETosis), in which the biological mechanism is mediated by ITIM-associated SHP-1. In short, these glycan-based immunomodulators may be applied in the future to treat KD patient refractory with IVIG resistance.
5. Targeting Signal Transduction
  Phosphoproteomics have shown that the overactivation of mitogen-activated kinase (MAPK) signaling cascades are involved in the pathogenesis of KD (25, 138). In addition, autoantibodies to MAPK and members of the casein kinase family (CSNK1A1, CSNK2A1, CSNK1E1) are also found in children with KD-like MIS-C (25). A synthetic lipid-modified sialoglycopeptide which inserts into cell membranes and mediates a cis-binding to Siglec-9 to inhibit MAPK activation, it has the potential for therapeutic suppression of autoimmune inflammation (139). Taken together, the specific targeting of the MAPK and CSNK pathways by protein kinase inhibitors, such as the modified sialoglycan that binds to Siglec-9, may be the key to anti-inflammatory treatment for the IVIG refractory KD.
6. Screening Genetic Variants and HLA Subtypes
  Polymorphisms of immune-related genes including HLA subtypes are critical to the etiology and severity of KD (40, 65–72). In Korea and Taiwan, the HLA-DRB1 is significantly associated with susceptibility of KD (67, 69), and we found that (MICA) A4 is a protective locus to protect KD patients from CAL (68). Recently, HLA-A02, B35, and C04 have been indicated to interact with superantigen-like motif of SARS-CoV-2 spike glycoprotein in MIS-C patients (140). In addition, genetic polymorphisms of the Treg pathway genes are associated with the susceptibility to KD (40). The gene expression of TLR1, 2, 4, 5, 8, and 9 are also involved in the pathogenesis of KD (74). To combine all the potential susceptible genes, we may be able to establish a specific genetic screening platform for the prediction of KD. This may help identify patients with a high risk of developing KD and offer early intervention and prevention of the disease.
7. Epigenetic Regulation of Treg Immune Responses
  Epigenetic modulations of DNA methylation and miRNA expression have been implicated in the pathogenesis of KD (73–77). Given the fact that T cell regulatory functions are affected by the epigenetic modulation of FoxP3, strategies for providing homeostasis of Treg immune responses might be applied to correct the Th17/Treg imbalance in complicated KD patients (78–81, 114– 117).
8. Inactivate Hemophagocytic Reactions
  Some patients with KDSS or MIS-C have evidence of MAS, in which laboratory studies revealed anemia, hyperferritinemia and hypertriglyceridemia. Macrophage activation is a result of the overexpression of Th1 mediators, such as IFN-γ and TNF-α, followed by the activation of Stat-1 and nuclear factor (NF)-κB. This pathophysiological response will lead to coagulopathy, hemophagocytosis and vasculitis (14, 16, 20, 21, 141, 142). The autoimmune-induced MAS is also occurring in patients with rheumatic diseases, including juvenile idiopathic arthritis, systemic lupus erythematosus and in patients with KD (41, 141, 143, 144). In this situation, a combination of IVIG with pulsed corticosteroids or administration of cyclosporin-A, anti-IL-1, and/or anti-TNF-α might be indicated (41, 97, 143–145).
  In summary, autoimmune vasculitis in KD and KDSS is mediated by a hyperinflammation response due to Th17/Treg imbalance, presumably associated with genetic variants of HLA, FcγRIIA, and/or epigenetic dysregulation. In the cases of IVIG resistant KD and KDSS, there are different phenotypes, susceptibility and immunopathogenesis, which in turn could be utilized for early prediction, prevention, and precision treatment. Regarding standard immunotherapies for KD, a consensus has been reached toward a combination of IVIG and aspirin with an additional pulse therapy of corticosteroids in high-risk patients. In refractory KD, individualized treatments such as anti-cytokine therapy or epigenetic induction should be considered to correct the Th17/Treg imbalance and cytokine storm hyperinflammation.

## Author Contributions

LC summarized the KD studies in Mackay Children Hospital for drafting the manuscript. H-WY drafted and revised the manuscript. T-YL collected the references and discussed the scheme for the writing of the manuscript. KY designed the article's outline and organized the information for the review, which has been approved by all authors before submission. All authors contributed to the article and approved the submitted version.

## Conflict of Interest

The authors declare that the research was conducted in the absence of any commercial or financial relationships that could be construed as a potential conflict of interest.
